# Psychometric properties of the Maslach Burnout Inventory for Medical Personnel (MBI-HSS-MP)

**DOI:** 10.1016/j.heliyon.2022.e08868

**Published:** 2022-02-01

**Authors:** Chung-Ying Lin, Zainab Alimoradi, Mark D. Griffiths, Amir H. Pakpour

**Affiliations:** a Institute of Allied Health Sciences, College of Medicine, National Cheng Kung University, Tainan, Taiwan; b Department of Public Health, College of Medicine, National Cheng Kung University, Tainan, Taiwan; c Department of Occupational Thearpy, College of Medicine, National Cheng Kung University, Tainan, Taiwan; d Social Determinants of Health Research Center, Research Institute for Prevention of Non-Communicable Diseases, Qazvin University of Medical Sciences, Qazvin 3419759811, Iran; e International Gaming Research Unit, Psychology Department, Nottingham Trent University, Nottingham, UK; f Department of Nursing, School of Health and Welfare, Jönköping University, Jönköping, Sweden

**Keywords:** Burnout, Factor analysis, Physician, Nurse, Validation

## Abstract

**Background:**

This study aimed to validate the Persian version of Maslach Burnout Inventory for Medical Personnel (MBI-HSS-MP), an instrument developed to capture burnout for health professionals. The specific aims were to psychometrically assess the Persian MBI-HSS-MP in relation to its structure, test-retest reliability, and item properties.

**Methods:**

The study setting was all eight hospitals in Qazvin province, Iran (study period from 10 September to 16 November 2020). Health professionals of physicians (n = 106) and nurses (n = 200) participated in the study. The psychometric properties of the 22-item MBI-HSS-MP was then examined for its factor structure via confirmatory factor analysis (CFA) and Rasch models, test-retest reliability, item fit, and differential item functioning (DIF).

**Results:**

The MBI-HSS-MP was verified as having a three-factor structure and each item was embedded well in its belonging construct (comparative fit index = 0.941, Tucker-Lewis index = 0.929 derived from CFA results; infit and outfit MnSq = 0.71 to 1.38 derived from Rasch models). Test-retest reliability of each MBI-HSS-MP item was satisfactory and no substantial DIF items were displayed across gender or across health professionals.

**Conclusion:**

The MBI-HSS-MP has good psychometric properties to assess burnout accurately among healthcare professionals in the three dimensions of emotional exhaustion, personal accomplishment, and depersonalization.

## Highlights

• The study confirmed the three-factor structure for the Maslach Burnout Inventory.
• Policymakers and managers can use this inventory to assess burnout and identify barriers.
• Different genders and health professionals interpret this inventory similarly.

## 1 Introduction

Burnout is defined as “a syndrome conceptualized as resulting from chronic workplace stress that has not been successfully managed” [1] in the 11th revision of the International Classification of Diseases (ICD-11). It is a syndrome comprising three dimensions (i.e., emotional exhaustion, personal accomplishment, and depersonalization). Emotional exhaustion refers to a human service worker who feels emotionally overextended and exhausted because of the human service work. Therefore, more emotional exhaustion is found among those with higher levels of burnout. Personal accomplishment refers to a human service worker who feels successful and competent when engaging in the human service work. Therefore, lower personal accomplishment is found among those with higher levels of burnout. Depersonalization refers to a human service worker who feels indifference and impersonal when providing human service to a service recipient. Therefore, stronger depersonalization is found among those with higher levels of burnout [2]. The Maslach Burnout Inventory (MBI), a commonly used psychometric instrument to assess burnout, also assesses the three dimensions of emotional exhaustion, personal accomplishment, and depersonalization. Moreover, there are different versions of the MBI that have been developed assessing specific or general populations (i.e., Medical Personnel, Human Services Workers, Educators, General Use, and Students) [2, 3, 4, 5, 6, 7, 8, 9, 10, 11, 12].

Several empirical studies have shown that different versions of MBI have satisfactory psychometric properties; for example, the three-factor structure has been supported in the Persian version of the MBI Human Services Workers (MBI-HSS) [6]. However, the Maslach Burnout Inventory for Medical Personnel (MBI-HSS-MP), a widely used instrument assessing burnout especially for health professionals has yet been examined for its psychometric properties in nurses and physicians [5]. In order to achieve the scientific rigor of a commonly used instrument (e.g., MBI-HSS-MP), one should always test for the instrument's psychometric properties to accumulate its scientific evidence to benefit the future users [13]. Therefore, it is important to use different methods of psychometric testing to support the robustness of the MBI-HSS-MP.

Burnout may lead to serious consequences if health professionals cannot cope with it [14, 15, 16]. Prior findings indicate that burnout among human services workers may decrease the quality of care or service because empirical evidence shows that burnout is an important factor associated with job turnover, low morale, and absenteeism among human service workers [14, 15, 16]. Therefore, patient safety may be at risk if health professionals (a type of human services) are influenced by burnout. Apart from the patient safety, health professionals experiencing burnout are at risk of various psychosocial problems, including personal dysfunction, physical exhaustion, sleep problems, overuse of alcohol and drugs, family arguments, and marital problems [5, 17, 18].

Given the importance of assessing burnout for health professionals in a precise way [5], empirical evidence of the psychometric properties for the Maslach Burnout Inventory for Medical Personnel (MBI-HSS-MP) [5], a widely used instrument assessing burnout especially for health professionals, is needed. Therefore, the present study utilized two types of psychometric testing to thoroughly understand the psychometric features of the MBI-HSS-MP among Iranian health professionals. More specifically, classical test theory (CTT) [19] and Rasch analysis [20] were used to evaluate the psychometric properties of the MBI-HSS-MP. The main benefit of using CTT is to provide the psychometric properties that most healthcare providers and researchers are familiar with [21, 22]. The main benefit of using Rasch analysis is to provide psychometric evidence based on the features of being sample-free [23]. CTT findings largely depend on the sample characteristics [19], while Rasch findings can minimize the effects of sample characteristics [20]. In addition to testing the psychometric properties of the MBI-HSS-MP using CTT and Rasch analysis, the present study applied latent profile analysis (LPA) [24] to classify the participants into different levels of burnout and examined whether health professionals with different levels of burnout have different characteristics.

In order to assess burnout among human service workers, the Maslach Burnout Inventory Human Services Survey (MBI-HSS) was developed using the three aforementioned dimensions of burnout [5]. The MBI-HSS was designed with an emphasis on staff-client interaction given that the nature of human services is spending considerable time intensely interacting with the service recipients [5]. The three-factor structure of the MBI-HSS (i.e., corresponding to the three dimensions of burnout) has been examined and supported among a variety of human services workers, including therapists [25], legal aid employees [5] and company employees [9]. Other psychometric features are also promising in the MBI-HSS. More specifically, the internal consistency of each MBI-HSS dimension is generally satisfactory, except for a slightly low Cronbach's α which was found in the depersonalization dimension [5]. Regarding the test-retest reliability, prior findings indicate that the MBI-HSS has a high degree of stability from one month to one year [5], which indicates that the MBI-HSS can capture an enduring psychological state. Prior psychometric evidence also shows that MBI-HSS has good convergent validity [3], discrimination validity [3, 6, 11], and predictive validity [10].

Although the MBI-HSS is a promising instrument to assess burnout for human services workers, it may not precisely capture the burnout for a specific type of human services workers (e.g., health professionals). Researchers and healthcare providers are extremely interested in understanding the issue of burnout among health professionals because this particular human service is important and related to human life. Indeed, a number of recent papers have discussed burnout among health professionals using the MBI-HSS [14, 15, 16, 17]. However, given that the service recipients of health professionals have unique features (i.e., they are usually ill and sick) from other types of service recipients, burnout among health professionals may be different from other human services workers. Therefore, the MBI-HSS-MP was developed to accurately assess burnout for health professionals.

Although the MBI-HSS-MP may share similar psychometric properties found in the MBI-HSS [4, 6, 10, 11], no psychometric evidence has been published concerning the MBI-HSS-MP [2]. The present study used different test theories (i.e., CTT and Rasch analysis) together to examine the psychometric properties of MBI-HSS-MP. If the psychometric properties of the MBI-HSS-MP can be supported by both CTT and Rasch analysis, such psychometric evidence can justify the accurate assessment of MBI-HSS-MP in relation to healthcare professions’ burnout. Therefore, the present study proposed the following hypotheses: (1) the MBI-HSS-MP will contain a three-factor structure; (2) each factor in the MBI-HSS-MP will be unidimensional; (3) different gender and different health professions will interpret the MBI-HSS-MP items in a similar way; and (4) the MBI-HSS-MP will have good known-group validity with the use of LPA.

## 2 Methods

### 2.1 Participants and procedure

The present methodological study was conducted between 10 September 2020 and 16 November 2020 in Qazvin, Iran. The study setting was all hospitals in Qazvin province. Study participants were physicians and nurses who were working in eight hospitals in Qazvin province. A convenience sampling approach was used to collect data from 400 physicians and nurses. After providing information on study aims and procedure, 94 physicians and nursed declined to participate in the study. The remaining 306 participants (106 physicians and 200 nurses), had a mean age of 45.12 years (SD = 6.42) and a larger proportion of females (62.4%).

The two types of health professions were recruited because of the following reasons: (1) nurses and physicians usually work in the same scenarios and contexts. Therefore, they may share some burnout factors similarly and it is important for researchers and policymakers to know if the MBI-HSS-MP can be applied to both nurses and physicians; and (2) when the psychometric features of the MBI-HSS-MP can be supported in the two types of health professionals, researchers, policymakers, and healthcare providers can use the MBI-HSS-MP scores to make further decisions regarding how to take care of burnout among these two types of health professionals. The participants were eligible to be included in the study if they worked full-time or part-time, providing direct care to patients, and provided written consent form to participate in the study.

### 2.2 Ethical consideration

The ethics committee of Qazvin University of Medical Sciences approved the study (ethical code: IR.QUMS.REC.1399.351). The study complies with all regulations and written informed consent was obtained.

### 2.3 Measures

#### 2.3.1 Maslach Burnout Inventory for Medical Personnel (MBI-HSS-MP)

The MBI-HSS-MP is a specific version of the MBI Human Services Survey. The MBI Human Services Survey was designed to assess how individuals working in human services feel exhausted or burnt out. However, given that medical personnel are one of the most studied occupational among all human services professions, the MBI-HSS-MP was further developed by slightly changing some of the wording from the MBI Human Services Survey. The MBI-HSS-MP contains 22 seven-point items distributed across three dimensions: Emotional Exhaustion (nine items), Personal Accomplishment (eight items), and Depersonalization (five items). The response scales are defined as follows: 0 = never, 1 = a few times a year or less, 2 = once a month or less, 3 = a few times a month, 4 = once a week, 5 = a few times a week, and 6 = every day. Higher scores indicate a higher level of burnout. The dimension scores can be calculated using two methods. The first method sums the item scores for each dimension and the second method calculates the average item scores for each dimension. The present study adopted the second method to calculate the MBI-HSS-MP dimension scores because this method provides a more straightforward interpretation of the dimension scores. More specifically, a mean dimension score of 3.5 can be interpreted as the participant feeling emotionally exhausted several times a month rather than emotionally exhausted every week. Psychometric properties of the MBI-HSS-MP have not been previously investigated. However, the MBI Human Services Survey has been tested among health professionals and has demonstrated a consistent three-factor structure [2, 5].

#### 2.3.2 Translation procedure of the MBI-HSS-MP into Persian

**Figure 1:**
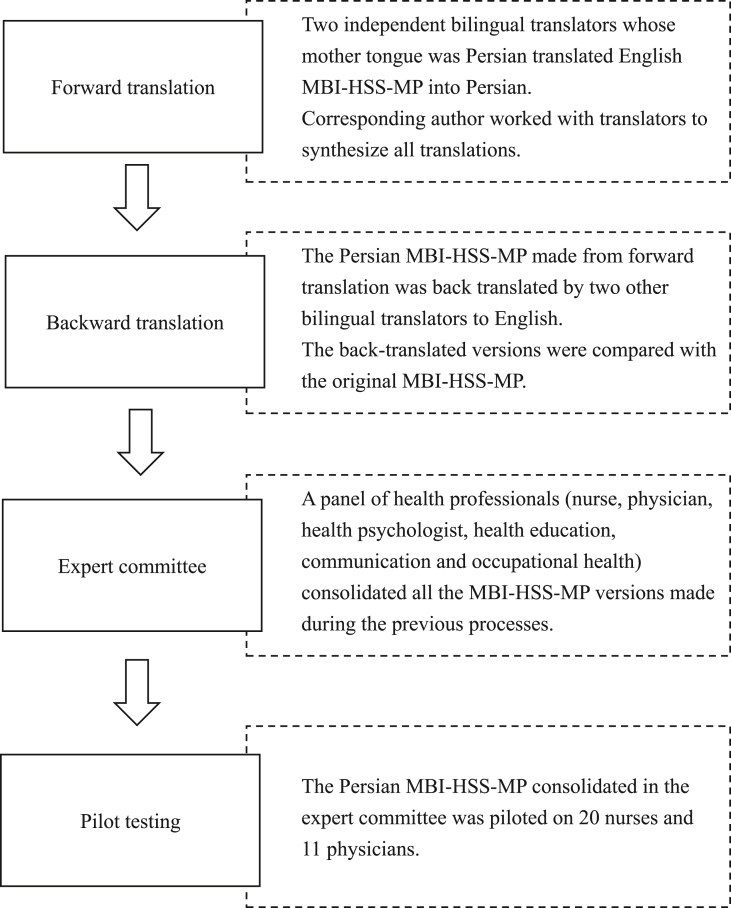
Flowchart of the present study.

The translation procedure was performed in several steps based on Beaton et al.‘s (2000) recommendations [26]. Moreover, the flowchart of the translation procedure is presented in Figure 1.

A. Forward translation

Two independent bilingual translators whose mother tongue was Persian, translated the English MBI-HSS-MP into Farsi/Persian. A session was then held with the corresponding author to synthesize all translations. The first stage attempted to develop an interim Persian version of the MBI-HSS-MP.

B. Backward translation

The interim Persian version was then translated back into English by two other bilingual translators who were blinded to the original English version. These back-translated versions were then compared with the original English versions.

C. Expert committee

A panel session with several health professionals (nurse, physician, health psychologist, health education, communication and occupational health) was held to consolidate all the versions of MBI-HSS-MP versions. The committee members assessed all translated items in terms of semantic equivalence, idiomatic equivalence, experiential equivalence, and conceptual equivalence.

D. Pilot testing

The pre-final Persian MBI-HSS-MP was then piloted on 20 nurses and 11 physicians to ensure that the target population could understand the items, responses, and instructions.

### 2.4 Data analysis

The demographics of the participants were analyzed using descriptive statistics before the psychometric testing of the MBI-HSS-MP was carried out. Two types of psychometric testing were utilized in the psychometric testing (i.e., CTT and Rasch analysis). Following this, LPA was used to classify the participants into different levels of burnout. Demographics and MBI-HSS-MP dimension scores were compared between the participants with different levels of burnout using analysis of variance (ANOVA).

Psychometric testing from CTT includes the following analyses. Normal distribution of each MBI-HSS-MP item score was assessed using skewness (an acceptable absolute value is below 3) and kurtosis (acceptable absolute value is below 10) [27]. Test-retest reliability of each item score was assessed using intraclass correlation coefficient (an acceptable value is above 0.7) [28]. Corrected item-total correlation of each item score was assessed with an acceptable value being above 0.4 [29]. Internal consistency of the MBI-HSS-MP dimension score was assessed using Cronbach's α and McDonald's ω (acceptable values are above 0.7) [30]. Composite reliability of the dimension score was assessed with an acceptable value above 0.6 [29]; average variance extracted of the dimension score was assessed with an acceptable value above 0.4 [31]. Moreover, confirmatory factor analysis (CFA) was used to examine whether the MBI-HSS-MP had a three-factor structure, a second-order factor structure (i.e., the three factors constructed into a higher order of latent construct), or a bifactor structure (i.e., the three specific factors together with a general factor). Several fit indices were applied to examine the fit for the CFA: comparative fit index (CFI) and Tucker-Lewis index (TLI) above 0.9; root mean square error of approximation (RMSEA) and standardized root mean square residual (SRMR) below 0.08 [32]. Akaike's information criterion (AIC) was used to determine which model performed better and a model with a lower AIC is a better model. In addition, factor loadings derived from the CFA were used to assess the MBI-HSS-MP item score with acceptable value above 0.4 [33].

Psychometric testing utilizing Rasch analyses included the following. Inlier-sensitive mean square (infit MnSq) and outlier-sensitive (outfit) MnSq were used to assess MBI-HSS-MP item score with acceptable value between 0.5 and 1.5 [34]. Differential item functioning (DIF) was used to assess whether participants of different gender and in different health professions interpreted the MBI-HSS-MP item in a similar way with acceptable absolute value of DIF contrast below 0.5 [35]. Item difficulties and item discriminations were calculated using the Rasch model. Moreover, item separation reliability and person separation reliability were used to assess the MBI-HSS-MP dimension score with acceptable value being above 0.7 [36]. Item separation index and person separation index were used to assess the MBI-HSS-MP dimension score with acceptable value being above 2 [37].

Latent profile analysis (LPA) was used to classify the participants into different levels of burnout according to their underlying latent structures of MBI-HSS-MP performance. With the use of goodness of fit, including AIC, Bayesian Information Criteria (BIC), and Sample Size Adjusted Bayesian Information Criterion (SSABIC), the LPA can help determine how many levels of burnout exist among the participants. More specifically, the lowest values of AIC, BIC, and SSABIC indicate better model fit and lead to the decision of group numbers [38]. Furthermore, entropy was used to assess the quality of model classification; a higher value of entropy indicates better classification. Lo-Mendell-Rubin's (LMR) likelihood ratio test was additionally used to understand whether every two types of grouping were significantly different from each other [39].

Regarding the statistics software, IBM SPSS AMOS was used for performing CFA; WINSTEPS for performing Rasch analyses; Mplus for performing LCA; IBM SPSS and Excel for performing the rest of the analyses.

## 3 Results

The participants were health professionals either as a physician (n = 106; 34.6%) or a nurse (n = 200; 65.4%). The mean age of the participants was 45.12 years (SD = 6.42) with nearly half of the sample having extensive clinical experience of over 10 years (49.0%). In addition, slightly more than one-third of the participants were males (37.6%) and most of the participants were married (88.2%) (Table 1).

**Table 1:** Participants characteristics (N = 306).

	Mean ± *SD* or n (%)
Age (Year)	45.12 ± 6.42
Gender (Male)	115 (37.6)
**Professional categories**	
Physician	106 (34.6)
Nurse	200 (65.4)
**Years of experience**	
<5	87 (24.8)
5–9	69 (22.5)
≥10	150 (49.0)
**Marital status**	
Single	24 (7.8)
Married	270 (88.2)
Divorced/widowed	12 (3.9)

Table 2 illustrates the item properties of the MBI-HSS-MP. The findings from CTT indicated that all the items had good factor loadings (values between 0.408 and 0.773), acceptable corrected item-total correlation (values between 0.294 and 0.666), satisfactory test-retest reliability (values between 0.70 and 0.92), and normal distributions (skewness = -1.80 to 1.10; kurtosis = -1.21 to 1.89). Findings from Rasch analyses indicate that all the items had good fit statistics (infit MnSq = 0.72 to 1.38; outfit MnSq = 0.71 to 1.36) and acceptable DIF (DIF contrasts = -0.30 to 0.20 across gender; DIF contrasts = -0.26 to 0.29 across health professions). In addition, the item difficulties were between -1.40 and 1.41; item discriminations were between 0.66 and 1.38.

**Table 2:** Psychometric properties of the Maslach Burnout Inventory Medical Personnel version at the item level.

Item#	Analyses from Classical Test Theory					Rasch Analyses					
	Factor loading∗^,^†	Item-total correlation	Test-retest reliability‡	S	K	Infit MnSq	Outfit MnSq	Difficulty	Discrimination	DIF contrast across gender§^,^¶	DIF contrast across professionals§^,^#
EE-1	0.637	0.605	0.86	0.30	-1.10	0.93	0.88	0.56	1.07	0.09	0.00
EE-2	0.497	0.468	0.75	-1.41	1.20	1.24	1.15	-1.40	0.92	0.20	0.20
EE-3	0.712	0.629	0.82	0.94	-0.14	0.75	0.71	-0.48	1.38	0.00	-0.05
EE-4	0.537	0.523	0.84	0.42	-1.21	1.13	1.17	0.62	1.02	0.03	-0.05
EE-5	0.746	0.666	0.80	-0.40	-0.58	0.77	0.80	-0.22	1.13	-0.12	0.08
EE-6	0.643	0.654	0.77	0.58	0.84	0.72	0.71	0.76	1.19	0.03	0.19
EE-7	0.495	0.388	0.75	-1.21	1.16	1.38	1.30	-1.10	0.71	-0.18	0.05
EE-8	0.513	0.523	0.73	1.10	-1.20	0.98	0.95	1.04	0.94	0.05	-0.06
EE-9	0.522	0.462	0.89	0.02	-0.39	1.26	1.25	0.22	0.70	-0.11	-0.04
PA-1	0.512	0.388	0.91	0.90	-1.07	1.02	1.19	-0.01	0.93	-0.21	0.22
PA-2	0.424	0.324	0.73	-0.30	-1.17	1.30	1.25	0.31	1.01	-0.03	-0.05
PA-3	0.658	0.553	0.70	-0.01	1.11	0.86	0.80	-0.50	1.12	-0.06	0.11
PA-4	0.530	0.508	0.78	1.01	-0.56	0.72	0.84	0.12	1.10	0.07	-0.26
PA-5	0.639	0.524	0.84	0.73	1.83	1.32	1.02	-0.36	1.13	-0.30	-.12
PA-6	0.773	0.596	0.82	-1.24	1.15	0.75	0.78	-0.23	1.12	-0.05	0.29
PA-7	0.718	0.545	0.80	-1.80	1.24	1.02	0.76	-0.74	1.15	-0.09	0.15
PA-8	0.408	0.294	0.75	-1.35	1.89	1.25	1.15	1.41	0.66	-0.25	-0.07
DP-1	0.637	0.521	0.92	-1.22	1.22	1.09	1.05	0.35	1.01	0.17	-0.22
DP-2	0.657	0.638	0.90	-1.04	0.75	0.77	0.76	-0.80	1.23	0.00	0.01
DP-3	0.504	0.557	0.83	-0.30	1.86	0.94	0.87	-0.67	1.02	-0.05	0.11
DP-4	0.583	0.452	0.85	-0.85	1.23	1.21	0.94	1.07	1.01	0.06	-0.07
DP-5	0.556	0.416	0.81	-1.15	-1.13	1.36	1.36	0.05	0.71	-0.12	-0.04

EE = Emotional Exhaustion; PA = Personal Accomplishment; DP = Depersonalization.

MnSq = mean square error; DIF = differential item functioning; S= Skewness; K= Kurtosis.

∗ All factor loadings were significant at 0.001.

† Based on the first-order confirmatory factor analysis (CFA). CFA model fit: χ^2^ (*df*) = 297.258 (206); comparative fit index = 0.941; Tucker-Lewis index = 0.929; root mean square error of approximation (90% CI) = 0.046 (0.033,0.058); and standardized root mean square residual = 0.069.

‡ Using Intraclass Correlation Coefficient (ICC).

§ DIF contrast >0.5 indicates substantial DIF.

¶ DIF contrast across gender = Difficulty for males-Difficulty for females.

# DIF contrast across gender = Difficulty for nurses-Difficulty for physicians.

The scale properties of the MBI-HSS-MP were also promising. In the CTT findings, Table 3 shows that the composite reliability (values between 0.73 and 0.83), average variance extracted (values between 0.35 and 0.36), and internal consistency (Cronbach's α between 0.733 and 0.844; McDonald's ω between 0.752 and 0.851) were acceptable or near acceptable. The Rasch analyses further support the good scale properties of the MBI-HSS-MP: item separation reliability between 0.99 and 1.00; item separation index between 8.33 and 16.27; person separation reliability between 0.73 and 0.85; person separation index between 2.15 and 2.34.

**Table 3:** Psychometric properties of the Maslach Burnout Inventory Medical Personnel version at the scale level.

Psychometric testing	EE	PA	DP
Composite Reliability	0.83	0.81	0.73
Average Variance Extracted	0.36	0.36	0.35
Internal consistency (Cronbach's α)	0.844	0.787	0.733
Internal consistency (McDonald's ω)	0.851	0.768	0.752
Item separation reliability from Rasch	1.0	0.99	0.99
Item separation index from Rasch	16.27	8.33	13.08
Person separation reliability from Rasch	0.85	0.73	0.78
Person separation index from Rasch	2.34	2.29	2.15

EE = Emotional Exhaustion; PA = Personal Accomplishment; DP = Depersonalization.

The CFA fit indices indicated that the all the three tested models were supported: CFI = 0.941, TLI = 0.929, RMSEA (90% CI) = 0.046 (0.033, 0.058), and SRMR = 0.069 for three-factor structure model; CFI = 0.915, TLI = 0.901, RMSEA (90% CI) = 0.055 (0.043, 0.066), and SRMR = 0.071 for second-order structure model; CFI = 0.920, TLI = 0.906, RMSEA (90% CI) = 0.054 (0.042, 0.065), and SRMR = 0.076 for bifactor structure model (Table 4). According to the AIC, the three-factor structure (AIC = 396.010) performed better than the second-order (AIC = 427.680) and the bifactor structure models (AIC = 423.683). The correlations between the three factors in the three-factor structure CFA model were 0.34 (between Emotional Exhaustion and Personal Accomplishment), 0.41 (between Personal Accomplishment and Depersonalization), and 0.66 (between Emotional Exhaustion and Depersonalization).

**Table 4:** Model comparison of different factor structures of the Maslach Burnout Inventory Medical Personnel version (MBI-HSS-MP).

Fit indices	MBI-HSS-MP		
	First-order model	Second-order model	Bifactor model
χ^2^ (df)	297.258 (206)	330.424 (206)	325.068 (206)
p-value	<0.001	<0.001	<0.001
CFI	0.941	0.915	0.920
TLI	0.929	0.901	0.906
RMSEA (90% CI)	0.046 (0.033, 0.058)	0.055 (0.043,0.066)	0.054 (0.042,0.065)
SRMR	0.069	0.071	0.076
AIC	396.010	427.680	423.683

CFI = comparative fit index; TLI = Tucker-Lewis index; RMSEA = root mean square error of approximation; SRMR = standardized root mean square residual; AIC = Akaike's information criterion.

The LPA suggested that the present sample should be classified into three groups (AIC = 24823.573, BIC = 25168.178, SSABIC = 24882.681, entropy = 0.942, and LMR test = 561.903; Table 5). According to the LPA, 67 participants were classified as low level of burnout, 80 as average level of burnout, and 159 as high level of burnout. Moreover, the demographic comparisons and MBI-HSS-MP dimension score comparisons are illustrated in Table 6. More specifically, the high level burnout group comprised health professionals of older age, more females, and longer years of experience, as compared with the low and average level burnout groups. Also, the high level burnout group reported higher levels of burnout across all three dimensions, as compared with the low and average level burnout groups.

**Table 5:** Latent profile analysis to identify subgroups of participants.

	AIC	BIC	SSABIC	Entropy	LMR test (*P*-value)
Profile 1	26493.448	26661.922	26522.346	n/a	n/a
Profile 2	25343.667	25600.207	25387.670	0.936	1186.928 (<0.0001)
Profile 3	**24823.573**	**25168.178**	**24882.681**	**0.942**	**561.903 (0.0010)**
Profile 4	24490.894	24923.565	24565.107	0.954	375.875 (0.1247)

AIC = Akaike's information criterion; BIC = Bayesian information criterion; SSABIC = sample-size adjusted BIC; LMR test = Lo-Mendell-Rubin's likelihood ratio test.

Bold values indicate that the nubmer of profile is the best fit.

**Table 6:** Predictors of membership in latent profile of risks internet addiction.

	1. Low burnout (n = 67)	2. Average burnout (n = 80)	3. High burnout (n = 159)	F (p-value)
Age in year	44.38 ± 4.33	46.09 ± 6.32	49.95 ± 6.69	5.221 (0.001)
Gender (Female)	32 (47.8%)	39 (48.7%)	120 (58.8%)	7.218 (0.027)
Years of experience	8.11 ± 5.01	10.16 ± 6.11	11.75 ± 5.75	5.541 (0.004)
Marital status (married)	54 (80.6%)	64 (80.0%)	152 (95.6%)	
Emotional Exhaustion score	3.11 ± 1.19	3.81 ± 1.14	4.08 ± 1.13	7.493 (<0.001)
Personal Accomplishment score	5.14 ± 0.67	4.95 ± 0.69	3.10 ± 0.54	8.521 (<0.001)
Depersonalization score	2.10 ± 1.14	2.24 ± 1.21	2.36 ± 1.25	7.116 (<0.001)

## 4 Discussion

To date, there has been a lack of psychometric evidence concerning the MBI-HSS-MP [5]. Consequently, the present study used data from 306 health professionals (i.e., nurses and physicians), to assess the psychometric properties of the MBI-HSS-MP. Two different psychometric testing methods (i.e., CTT and Rasch analysis) were applied and both methods indicated that the MBI-HSS-MP has promising properties for healthcare providers or researchers to accurately and precisely assess burnout among health professionals. Moreover, the LPA conducted in the present study found that the present sample could be classified into three different levels of burnout. Different features of participants with different levels of burnout were demonstrated.

Given that there is currently no psychometric evidence for the MBI-HSS-MP in the extant literature, comparison of the present study's findings to other MBI-HSS-MP evidence is not possible. However, the psychometric properties of the MBI-HSS-MP in the present study can be compared with the prior psychometric findings on the MBI-HSS. Prior psychometric findings on the MBI-HSS indicated good internal consistency concerning each MBI-HSS dimension, except for a slightly low Cronbach's α in the depersonalization dimension [5]. The present study found that all three MBI-HSS-MP dimensions, which comprise the same three dimensions as the MBI-HSS, were satisfactory. Additionally, prior studies have reported that the MBI-HSS has a high degree of stability from one month to one year [2]. The present study extended the prior test-retest findings to the item levels and found that the test-retest reliability is satisfactory not only for the three MBI-HSS-MP dimensions, but also for all 22 MBI-HSS-MP items. However, different factor structures and issues concerning the Maslach Burnout Inventory (MBI) require further discussion. For example, two to four factors have been proposed for the MBI among educators [12]. Moreover, some researchers have proposed a potential wording effect in the MBI [4] and such wording effects may interfere with the factor structure of the MBI. The MBI General Survey was found to be a three-factor structure but the three factors (i.e., exhaustion, cynicism, and professional efficacy) are different from the MBI-HSS (i.e., emotional exhaustion, personal accomplishment, and depersonalization) [7, 8]. Therefore, additional studies are needed to consolidate evidence regarding the factor structure of the MBI-HSS-MP.

Although factor structure of MBI-HSS-MP may need more evidence, the present study's findings show that the MBI-HSS-MP has strong psychometric properties at both item and scale levels. At the item level, normal distributions were identified for all the items. Moreover, all the items fitted in their correct dimensions. Therefore, the three types of burnout (i.e., emotional exhaustion, personal accomplishment, and depersonalization [5]) are accurately quantified by the MBI-HSS-MP items. Satisfactory test-retest reliability of the MBI-HSS-MP items suggest the stability of each item. Additionally, no substantial DIF was displayed for any MBI-HSS-MP items, which implies that different subgroups in the present sample (i.e., gender and profession group) share the same interpretations toward all the MBI-HSS-MP items [23, 35].

At the scale level, all three models (i.e., the three-factor structure, the second-order structure, and the bifactor structure) of the MBI-HSS-MP were confirmed by the CFA. However, the fit indices were only borderline satisfactory (i.e., between 0.9 and 0.95 for CFI and TLI). According to the latest recommendation, CFI and TLI should be higher than 0.95 [40]. Moreover, the three factor-structure of the MBI-HSS-MP performed better than the other two models (i.e., the second-order structure and the bifactor structure). Therefore, the conceptualization of three types of burnout [5] was again supported. The reliability of the MBI-HSS-MP was supported by different psychometric methods, including internal consistency, composite reliability, average variance extracted, Rasch item separation reliability, Rasch item separation index, Rasch person separation reliability, and Rasch person separation index. These promising reliability findings indicate that the MBI-HSS-MP can effectively assess and distinguish health professionals at different levels of burnout. Indeed, the LPA findings indicate that there are three levels of burnout. Moreover, reliability findings from the Rasch model indicate that the satisfactory psychometric properties of the MBI-HSS-MP are not influenced by the present sample's characteristics [23, 35].

There are some limitations in the present study. First, there were no external outcome measures. Therefore, the convergent validity and discrimination validity of the MBI-HSS-MP was only examined using the CFA rather than using external outcome measures. Future studies may consider using different outcome measures (e.g., quality of life measures) to provide additional validity evidence for the MBI-HSS-MP. Second, only two types of health professional (i.e., physicians and nurses) were recruited in the present study. Therefore, the present findings cannot necessarily be generalized to other types of health professional. Future studies may want to examine the psychometric properties of the MBI-HSS-MP among other types of health professional (e.g., occupational therapists, physiotherapists, podiatrists, optometrists, etc.). Third, all the participants were Iranians and the present findings cannot be generalized to other ethnic groups. Fourth, the present sample size was relatively small, and this can sometimes be a limitation in advanced psychometric testing such as CFA. Finally, all the participants were in full-time work employment, therefore the findings cannot necessarily be generalized to those in part-time work employment. Future studies could therefore investigate measurement invariance comparison based on type of employment.

## 5 Implications

Given that the psychometric properties of the MBI-HSS-MP were rigorously examined and produced satisfactory results, clinical and nursing practitioners may use the MBI-HSS-MP to assess the levels of burnout among health professionals. Subsequently, early treatments or interventions can be applied to prevent serious health problems caused by burnout among those in the health profession. More specifically, the present findings have nursing implications in the following aspects. First, healthcare providers’ burnout can be assessed in a better and more accurate way according to the satisfactory psychometric properties found in the present study. Consequently, this could be helpful for improving mental health among those in the nursing profession. Second, the results of the present study can be used to better understand latent profiles of nursing staff with any level of burnout. That is, the findings support the use of the MBI-HSS-MP in the early detection of the initial stage of burnout among nursing staff. This could also be helpful for improving mental health among nursing professionals. Third, it is estimated that over 120 million people speak Persian worldwide (i.e., individuals residing in Iran, Afghanistan, Tajikistan, and Uzbekistan). Therefore, the results of the study can especially assess burnout among Persian-speaking nursing staff. Finally, the results of the present study can be used by policymakers and managers to assess burnout and to identify barriers to improve nursing mental health at the governmental level.

## 6 Conclusion

In conclusion, the present study demonstrated the promising psychometric properties of the MBI-HSS-MP, an instrument for assessing burnout among health professionals. The factor structure of the MBI-HSS-MP was verified to be a three-factor structure and all the items in each factor fitted their embedded constructs well. Therefore, in future studies, researchers and healthcare providers may use MBI-HSS-MP to assess three dimensions of burnout (emotional exhaustion, personal accomplishment, and depersonalization) among health professionals.

## Declarations

### Author contribution statement

Chung-Ying Lin: Conceived and designed the experiments; Analyzed and interpreted the data; Contributed reagents, materials, analysis tools or data; Wrote the paper.

Zainab Alimoradi: Performed the experiments; Contributed reagents, materials, analysis tools or data; Wrote the paper.

Mark D. Griffiths: Analyzed and interpreted the data; Wrote the paper.

Amir H Pakpour: Conceived and designed the experiments; Performed the experiments; Analyzed and interpreted the data; Contributed reagents, materials, analysis tools or data; Wrote the paper.

### Funding statement

This research did not receive any specific grant from funding agencies in the public, commercial, or not-for-profit sectors.

### Data availability statement

Data will be made available on request.

### Declaration of interests statement

The authors declare no conflict of interest.

### Additional information

No additional information is available for this paper.

## References

[1] World Health Organization (2018).

[2] Maslach C., Jackson S., Leiter M., Schaufeli W., Schwab R. (2018).

[3] Calderón-de la Cruz G.A., Merino-Soto C., Juárez-García A., Dominguez-Lara S., Fernández-Arata M. (2020). Is the factorial structure of the Maslach Burnout Inventory Human Service Survey (MBI-HSS) replicable in the nursing profession in Peru? A national study. Enfermería Clínica (English Edition).

[4] Gómez García R., Alonso Sangregorio M., Lucía Llamazares Sánchez M. (2019). Factorial validity of the Maslach Burnout Inventory-human services survey (MBI-HSS) in a sample of Spanish social workers. J. Soc. Serv. Res..

[5] Lheureux F., Truchot D., Borteyrou X., Rascle N. (2017). The Maslach Burnout Inventory–Human Services Survey (MBI-HSS): factor structure, wording effect and psychometric qualities of known problematic items. Trav. Hum. Le..

[6] Moalemi S., Kavoosi Z., Beygi N., Deghan A., Karimi A., Parvizi M.M. (2018). Evaluation of the Persian version of maslach burnout inventory-human services survey among Iranian nurses: validity and reliability. Galen Med. J..

[7] Schutte N., Toppinen S., Kalimo R., Schaufeli W. (2000). The factorial validity of the Maslach Burnout Inventory-General Survey (MBI-GS) across occupational groups and nations. J. Occup. Organ. Psychol..

[8] Taris T.W., Schreurs P.J., Schaufeli W.B. (1999). Construct validity of the Maslach Burnout Inventory-General Survey: a two-sample examination of its factor structure and correlates. Work. Stress.

[9] da Silva Valente MdS., Wang Y.-P., Menezes P.R. (2018). Structural validity of the maslach burnout inventory and influence of depressive symptoms in banking workplace: unfastening the occupational conundrum. Psychiatr. Res..

[10] Wickramasinghe N.D., Dissanayake D.S., Abeywardena G.S. (2018). Clinical validity and diagnostic accuracy of the Maslach burnout inventory-student survey in Sri Lanka. Health Qual. Life Outcome.

[11] Wickramasinghe N.D., Dissanayake D.S., Abeywardena G.S. (2018). Validity and reliability of the maslach burnout inventory-student survey in Sri Lanka. BMC Psychol..

[12] Worley J.A., Vassar M., Wheeler D.L., Barnes L.L. (2008). Factor structure of scores from the Maslach Burnout Inventory: a review and meta-analysis of 45 exploratory and confirmatory factor-analytic studies. Educ. Psychol. Meas..

[13] Lin C.-Y., Hwang J.-S., Wang W.-C. (2019). Psychometric evaluation of the WHOQOL-BREF, Taiwan version, across five kinds of Taiwanese cancer survivors: Rasch analysis and confirmatory factor analysis. J. Formos. Med. Assoc..

[14] Dyrbye L.N., Shanafelt T.D., Johnson P.O., Johnson L.A., Satele D., West C.P. (2019). A cross-sectional study exploring the relationship between burnout, absenteeism, and job performance among American nurses. BMC Nurs..

[15] Nocon R.S., Fairchild P.C., Gao Y. (2019). Provider and staff morale, job satisfaction, and burnout over a 4-year medical home intervention. J. Gen. Intern. Med..

[16] Scanlan J.N., Still M. (2019). Relationships between burnout, turnover intention, job satisfaction, job demands and job resources for mental health personnel in an Australian mental health service. BMC Health Serv. Res..

[17] Lichstein P.M., He J.K., Estok D. (2020). What is the prevalence of burnout, depression, and substance use among orthopaedic surgery residents and what are the risk factors? A Collaborative Orthopaedic Educational Research Group survey study. Clin. Orthop. Relat. Res..

[18] Papaefstathiou E., Apostolopoulou A., Papaefstathiou E., Moysidis K., Hatzimouratidis K., Sarafis P. (2020). The impact of burnout and occupational stress on sexual function in both male and female individuals: a cross-sectional study. Int. J. Impot. Res..

[19] DeVellis R.F. (2006). Classical test theory. Med. Care.

[20] Bond T.G., Fox C.M. (2013).

[21] Lin C.-Y., Pakpour A.H., Broström A. (2018). Psychometric properties of the 9-item European Heart Failure Self-Care Behavior Scale using confirmatory factor analysis and Rasch analysis among Iranian patients. J. Cardiovasc. Nurs..

[22] Lin C.-Y., Oveisi S., Burri A., Pakpour A.H. (2017). Theory of Planned Behavior including self-stigma and perceived barriers explain help-seeking behavior for sexual problems in Iranian women suffering from epilepsy. Epilepsy Behav..

[23] Chang C.-C., Su J.-A., Tsai C.-S., Yen C.-F., Liu J.-H., Lin C.-Y. (2015). Rasch analysis suggested three unidimensional domains for Affiliate Stigma Scale: additional psychometric evaluation. J. Clin. Epidemiol..

[24] Flaherty B.P., Kiff C.J. (2012).

[25] Al Mutair A., Al Mutairi A., Chagla H., Alawam K., Alsalman K., Ali A. (2020). Examining and adapting the psychometric properties of the Maslach burnout inventory-health services survey (MBI-HSS) among healthcare professionals. Appl. Sci..

[26] Beaton D., Bombardier C., Guillemin F., Ferraz M.B. (2002). Recommendations for the cross-cultural adaptation of health status measures. Am. Acad. Orthopaed. Surg..

[27] Lin C.-Y., Luh W.-M., Yang A.-L., Su C.-T., Wang J.-D., Ma H.-I. (2012). Psychometric properties and gender invariance of the Chinese version of the self-report pediatric quality of life inventory version 4.0: short form is acceptable. Qual. Life Res..

[28] Lin Y.-C., Strong C., Tsai M.-C., Lin C.-Y., Fung X.C. (2018). Validating sizing them up: a parent-proxy weight-related quality-of-life measure, with community-based children. Int. J. Clin. Health Psychol..

[29] Lin C.-Y., Griffiths M.D., Pakpour A.H. (2018). Psychometric evaluation of Persian Nomophobia Questionnaire: differential item functioning and measurement invariance across gender. J. Behav. Add..

[30] Cheng C.-P., Luh W.-M., Yang A.-L., Su C.-T., Lin C.-Y. (2016). Agreement of children and parents scores on Chinese version of pediatric quality of life inventory version 4.0: further psychometric development. Appl. Res. Qualit. Life.

[31] Huang C.-C., Wang Y.-M., Wu T.-W., Wang P.-A. (2013). An empirical analysis of the antecedents and performance consequences of using the moodle platform. Int. J. Inform. Educ. Techn..

[32] Chen I.-H., Strong C., Lin Y.-C. (2020). Time invariance of three ultra-brief internet-related instruments: smartphone application-based addiction scale (SABAS), Bergen social media addiction scale (BSMAS), and the nine-item internet gaming disorder scale-short form (IGDS-SF9)(study Part B). Addict. Behav..

[33] Nejati B., Lin C.-Y., Griffiths M.D., Pakpour A.H. (2020). Psychometric properties of the Persian Food-Life Questionnaire Short Form among obese breast cancer survivors. Asia-Pac. J. Oncol. Nurs..

[34] Nejati B., Fan C.-W., Boone W.J., Griffiths M.D., Lin C.-Y., Pakpour A.H. (2020). Validating the Persian Intuitive Eating Scale-2 among breast cancer survivors who are overweight/obese. Eval. Health Prof..

[35] Lin C.-Y., Broström A., Griffiths M.D., Pakpour A.H. (2020). Psychometric evaluation of the Persian eHealth Literacy Scale (eHEALS) among elder Iranians with heart failure. Eval. Health Prof..

[36] Lin C.-Y., Imani V., Griffiths M.D., Pakpour A.H. (2021). Validity of the yale food addiction scale for children (YFAS-C): classical test theory and item response theory of the Persian YFAS-C. Eating Weight Disord. Stud. Anorex. Bulimia Obes..

[37] Imani V., Lin C.-Y., Jalilolgadr S., Pakpour A.H. (2018). Factor structure and psychometric properties of a Persian translation of the epworth sleepiness scale for children and adolescents. Health Promot. Perspect..

[38] Lin C.-Y., Imani V., Broström A. (2019). Smartphone application-based addiction among Iranian adolescents: a psychometric study. Int. J. Ment. Health Addiction.

[39] Tein J. (2013). Statistical power to detect the correct number of classes in latent profile analysis. Struct. Equ. Model..

[40] Kline R. (2016).

